# Selection Signature Analyses Revealed Genes Associated With Adaptation, Production, and Reproduction in Selected Goat Breeds in Kenya

**DOI:** 10.3389/fgene.2022.858923

**Published:** 2022-04-21

**Authors:** Ruth W. Waineina, Tobias O. Okeno, Evans D. Ilatsia, Kiplangat Ngeno

**Affiliations:** ^1^ Department of Animal Sciences, Animal Breeding and Genomics Group, Egerton University, Egerton, Kenya; ^2^ Dairy Research Institute, Kenya Agricultural and Livestock Organization, Naivasha, Kenya

**Keywords:** adaptation, candidate genes, footprints, signature of selection, putative regions

## Abstract

Artificial and natural selection in livestock is expected to leave unique footprints on their genomes. Goat breeds in Kenya have evolved for survival, breeding, and production in various harsh ecological areas, and their genomes are likely to have acquired unique alleles for adaptation to such diverse production environments and other traits of economic importance. To investigate signals of selection for some selected goat breeds in Kenya, Alpine (*n* = 29), Galla (*n* = 12), Saanen (*n* = 24), and Toggenburg (*n* = 31) were considered. A total of 53,347 single-nucleotide polymorphisms (SNPs) generated using the Illumina GoatSNP50 BeadChip were analyzed. After quality control, 47,663 autosomal single-nucleotide polymorphisms remained for downstream analyses. Several complementary approaches were applied for the following analyses: integrated Haplotype Score (iHS), cross-population-extended haplotype homozygosity (XP-EHH), hapFLK, and FLK. A total of 404 top genomic regions were identified across all the four breeds, based on the four complementary analyses. Out of the 16 identified putative selection signature regions by the intersection of multiple-selective signal analyses, most of the putative regions were found to overlap significantly with the iHS and XP-EHH analyses on chromosomes 3, 4, 10, 15, 22, and 26. These regions were enriched with some genes involved in pathways associated directly or indirectly with environmental adaptation regulating immune responses (e.g., *HYAL1* and *HYAL3*)*,* milk production (e.g., *LEPR* and *PDE4B*), and adaptability (e.g., *MST1* and *PCK*). The results revealed few intersect between breeds in genomic selection signature regions. In general, this did not present the typical classic selection signatures as predicted due to the complex nature of the traits. The results support that some various selection pressures (e.g., environmental challenges, artificial selection, and genome admixture challenges) have molded the genome of goat breeds in Kenya. Therefore, the research provides new knowledge on the conservation and utilization of these goat genetic resources in Kenya. In-depth research is needed to detect precise genes connected with adaptation and production in goat breeds in Kenya.

## Introduction

Goats were the first ruminant animal to be domesticated from wild bezoar goat (*Capra aegagrus*) in the ancient civilization along the rivers of Nile (Africa), Tigris and Euphrates (Asia), and Indus (India) (Naderi et al., 2007). Domestication occurred about 10,000 years ago (Zeder and Hesse, 2000). They spread all over the world, including Africa, following the pattern of human migrations with their livestock and trade routes (Luikart et al., 2001; Taberlet et al., 2008). In the process of migrations, the goats adapted to a wide range of various geographical conditions, ranging from the mountains to the desert and tropical regions of Africa.

Goat populations were managed differently, with both natural and artificial selection playing a major role in their genetic variation. In the process of domestication, selection, and breed formation, some regions within the goat genomes may have developed special patterns to reflect such development. According to Smith and Haigh (1974), positive selection pressure increases the frequency of beneficial alleles, leaving unique signatures in the genome. In an organized screening of the genome for genes under selection, these exceptional signatures can be utilized by identifying genomic regions or loci showing deviations from neutrality. Apart from selection, other factors influence selection signatures, for example, the strength of selection, the relative age of the neutral-linked alleles, and the recombination rate (Kim and Stephan, 2002; Charlesworth, 2007; McVean, 2007).

In Kenya, the goat population is estimated to be about 26.7 million head distributed in all the agroecological zones (FAO STAT, 2018). Dairy goat populations, mainly exotic breeds such as Toggenburg, Anglo-Nubian, German Alpines, Saanen, and Boer were first introduced in Kenya in the 1950s by the British settler farmers (Shivairo et al., 2013). Succeeding introductions were through the collaboration between the Government of Kenya (GoK) with donor agencies or by non-governmental organizations (NGOs). They were introduced mainly for crossbreeding with local populations to improve both the milk and growth performance of the indigenous goats (Ahuya et al., 2009; Bett et al., 2011; Shivairo et al., 2013). Among the exotic goat breeds in Kenya, this study concentrated on Toggenburg, Saanen, and Alpine breeds. The breeds are reared in intensive and semi-intensive production systems based on agroecological zones and management regimes. The main breeding objective for Toggenburg and Saanen was milk production whereas for the Alpine breed is breeding flock.

Indigenous goats (e.g., Galla and small East Africa) and the crossbred of exotic and indigenous are known to be well adapted to various ecological areas in Kenya as shown by their better performance achievement (Ahuya et al., 2009; Ndeke et al., 2015; Kiura et al., 2020). Their characteristics such as resistance to dehydration, preference for browsing, and a wide range of feeding habits (Chenyambuga et al*.* 2004) have allowed them to adapt to the expanse of arid and semi-arid regions in the country. The Galla goat breed in this study was introduced in the Naivasha Sheep and Goats Station in the early 1970s during a sheep and goat project funded by the FAO (Pillian and Racokzi, 1976). They are maintained in this government breeding and conservation station as a pure breed for meat and milk production and to reproduce under harsh conditions in an extensive production system.

The phenotypic characteristics of these goats since introduction have been determined by the adaptation to new environments and artificial selection based on distinct breeding goals. Artificial selection increases the beneficial alleles associated with economic traits and helps in improving the production factors. Putative loci in the genome that controls these traits in the four goat breeds in Kenya have not been identified so far, which has slowed down the understanding of the mechanism of selection in these goats. It can therefore be assumed that these selected goats have evolved for survival, breeding, and production in various ecological areas since their genomes have footprints for adaptation to such environment and other traits of economic importance.

In goats, selection signature analyses using genome-wide SNPs have been used in discovering their genomes (Wang et al., 2016; Brito et al., 2017; Onzima et al., 2018). The approach has identified several putative genomic candidate regions and genes under selection that influenced some traits such as thermo-tolerance, energy and digestive metabolism, nervous and autoimmune response, body size and development, skin and hair structure, and reproduction and functions.

Many statistical approaches have been developed to identify footprints of selection in recent years. These approaches are generally classified into three key groups 1) the linkage disequilibrium (LD)-based approach (e.g., integrated Haplotype Score (iHS), cross-population-extended haplotype homozygosity (XP-EHH), and hapFLK), 2) site frequency spectrum (SFS) (e.g., *Tajima’s D* and *Fu and Li’s tests*), and 3) population differentiation-based tests (e.g., FLK) (Weigand and Leese, 2018). To detect the signatures of selection that exploit the benefit of complementarity and to improve the statistical power, several studies have been conducted exploiting more than one test statistic.

In this study, four complementary approaches were applied as follows: iHS, FLK, hapFLK, and XP-EHH. When a good reference population cannot be found, the iHS test is anticipated to be more reliable. However, it has low power when the selected allele is close to fixation (Voight et al., 2006). According to the primary evolutionary version, the FLK statistic assumes that SNPs were already in polymorphic in the ancestral breeds, and hapFLK is robust when considering migration and bottlenecks. The XP-EHH statistic evaluates haplotype differences between two potentially divergent populations because it is aimed to identify alleles that have increased in frequency to the point of fixation or near fixation in one of the populations (Sabeti et al., 2007). The objective of this study was to identify distinctive selection signatures in the genome and the genes mapping to these candidate regions of goat breeds in Kenya.

## Materials and Methods

### Ethics Statement

The study was conducted in strict accordance with the recommendations from the Institute of Primate Research (IPR) ethical guidelines on Animal Care and Use of Laboratory Animals. The protocol was approved by the Committee on the Ethics of Animal Experiment of Egerton University of Egerton in Kenya (ISERC/03/2020). A qualified veterinary officer collected the whole blood following FAO guidelines (FAO, 2012) to reduce pain and discomfort to a minimum.

### Animals and Genotype Control

Data used were obtained from 96 samples across four goat breeds. The four breeds were Galla (*n* = 12), Alpine (*n* = 29), Saanen (*n* = 24), and Toggenburg (*n* = 31). Animals were genotyped using the Illumina GoatSNP50 BeadChip (Tosser-Klopp et al., 2014) containing 53,347 single-nucleotide polymorphisms (SNPs). Quality control (QC) on genotype data was performed using PLINK v1.90 (Chang et al., 2015). The genotyping rate was 0.979687. Out of the 96 samples, two samples failed due to missing genotype data (--mind 0.05); 1,079 variants were removed due to missing genotype data (--geno 0.1); 527 variants were removed due to the Hardy–Weinberg exact test (--hwe 0.001), and 1,305 variants were removed due to a minor allele frequency threshold (--maf 0.05). Additionally, variants located on non-autosomal chromosomes were discarded. The genotyping rate in the remaining 94 samples was 0.98088. Following these QC procedures, 94 individuals and a total of 47,663 SNPs (with a genotyping rate of 0.98) remained for downstream analysis.

### Identifying Selection Signatures

A total of four goat breeds were considered as four different populations in the signature of selection analyses, namely, Alpine, Galla, Saanen, and Toggenburg. Various methods have been used to improve the likelihood to detect true selection signatures (i.e., no false-positive results) (Qanbari and Simianer, 2014). The following are the various methods that were applied in detecting selection signatures. Several different methods were used to exploit the benefit of complementarity and to improve the statistical power.

An iHS was estimated using *fast*PHASE and performed using the *rehh* package (Gautier and Vitalis, 2012) in R v. 3.4.4. Following analyses, the |iHS| values were transformed into −log 10 [1–2|Φ iHS −0.5|], in which Φ iHS is the cumulative Gaussian distribution function for better visualization and assessment of selection signals. According to Gautier and Naves (2011), assuming a normal distribution, |iHS| values can be interpreted as log10 (1/P), where P is a one-sided *p*-value associated with the neutral hypothesis of no selection. In this study, all the SNPs ranking above 0.1 percentile of the |iHS| distribution were selected as candidates for selection. This is a conservative cutoff value corresponding to *p*-value < 0.00001 and a false discovery rate (FDR) threshold of less than 0.01 following the formula described by Bolormaa et al. (2011). This cutoff threshold was chosen based on the fact that the sample size used was small (*n* = 94) and in a bid to guard against false-positives.

To detect potential selection signatures of main differentiation between four goat breeds, the FLK (Bonhomme et al., 2010) and hapFLK (Fariello et al., 2013) methods were applied. The FLK statistic was analyzed for each SNP within each breed. The hapFLK method was also used to account for haplotype structure and changing population sizes. The hapFLK method used unphased data of multiple populations to determine cluster identities. These identities were then used to calculate a population differentiation statistic, which incorporated a kinship matrix, representing the relationship between breeds based on Reynolds genetic distance. For each SNP, the pairwise Reynolds genetic distances among populations were calculated and averaged over the genome. Genotype data, kinship matrix, and assumption of four clusters in the fastPHASE model (Scheet and Stephens, 2006) (−k, 4) were used to run the program. To fit the linkage disequilibrium (LD) model, the hapFLK statistic was calculated as an average of 20 expectation maximization iterations. The *p-*values were estimated based on a chi-square distribution of the numerical values using the hapFLK values generated for each SNP as described by Fariello et al. (2013). As in the iHS test, candidate selection SNPs were defined as those ranking the top 0.1 percentile distribution of hapFLK and FLK–log (*p*-values) corresponding to *p* < 0.01.

In total, three exotic goats breed (Saanen, Alpine, and Toggenburg) genomes were used as a test population with the genome of Galla used as a reference population to identify positive selection signatures in exotic goat populations. Several goat comparisons were performed using the XP-EHH method (Sabeti et al., 2007) as follows: Alpine versus Galla; Saanen versus Alpine; Saanen versus Galla; and Toggenburg versus Galla. The XP-EHH is designed to detect SNPs that are under selection in one population but not in another. Basically, it compares the haplotype homozygosity (EHH) and integrated Haplotype Score (iHS) among the populations. Therefore, XP-EHH analyses consider distinct SNPs amongst populations that are homozygous for one and polymorphic for others using the comparison of the EHH score of two populations. A positive XP-EHH value indicates selections occurred in the test population, while a negative value indicates selection in the reference/control population. The XP-EHH was calculated as follows:

$$XP-EHH=In\;\left(\frac{I_{A}}{I_{B}}\right),$$

where *I*_*A*_ is the integrated value of the test population EHH, and *I*_*B*_ is the integrated value of the reference population EHH.

For visualization and interpretation of regions under selection, the XP-EHH scores were standardized to a distribution with zero mean and unit variance. As in previous signatures of selection analyses, SNPs ranking top 0.1 percentile were considered a candidate for selection from XP-EHH analyses. This cutoff threshold corresponds to a different *p*-value for different population comparisons: Alpine vs. Galla (*p* < 0.001); Saanen vs. Alpine (*p* < 1.0e-05); Toggenburg vs. Galla (0.001), and Saanen vs. Galla (*p* < 1.0e-04).

### Candidate Genes Associated With Selection Signature Identification

Based on the aforementioned results, for all the SNPs passing the top 0.1 percentile threshold from all the four methods, intersection of multiple-selective signal analysis was performed. In the regions which overlapped across the analysis, goat gene mapping was extracted to 10 kb up-downstream for each significant SNP using the BioMart tool based on the goat reference genome assembly (ARS1). The protein-coding genes which overlapped with the regions under positive selection were defined as candidate genes. Gene enrichment analyses were conducted using the database for annotation, visualization, and integrated discovery (DAVID) v6.8 (Sherman and Lempicki, 2009), which permits for the investigation of the Kyoto Encyclopedia of Genes and Genomes (KEGG) pathway (Kanehisa et al., 2012) and Gene Ontology (GO) for biological processes (Ashburner et al., 2000). To identify significantly enriched GO biological process, molecular function, and cellular component, Fischer’s exact test (*p*-value = 0.05) was applied. Due to the limited scope of the study, more rigorous settings, such as Bonferroni correction and Fold enrichment test, were not considered in the detection. Human gene ontologies were used because the goat genome has not been properly annotated. In addition, the human genome is well annotated than that of other species, therefore improving the probability of retrieving GO terms in the goat genome. Finally, an extensive review of the literature to annotate functions of the identified genes was performed.

## Results

### Selection Signatures

**FIGURE 1:**
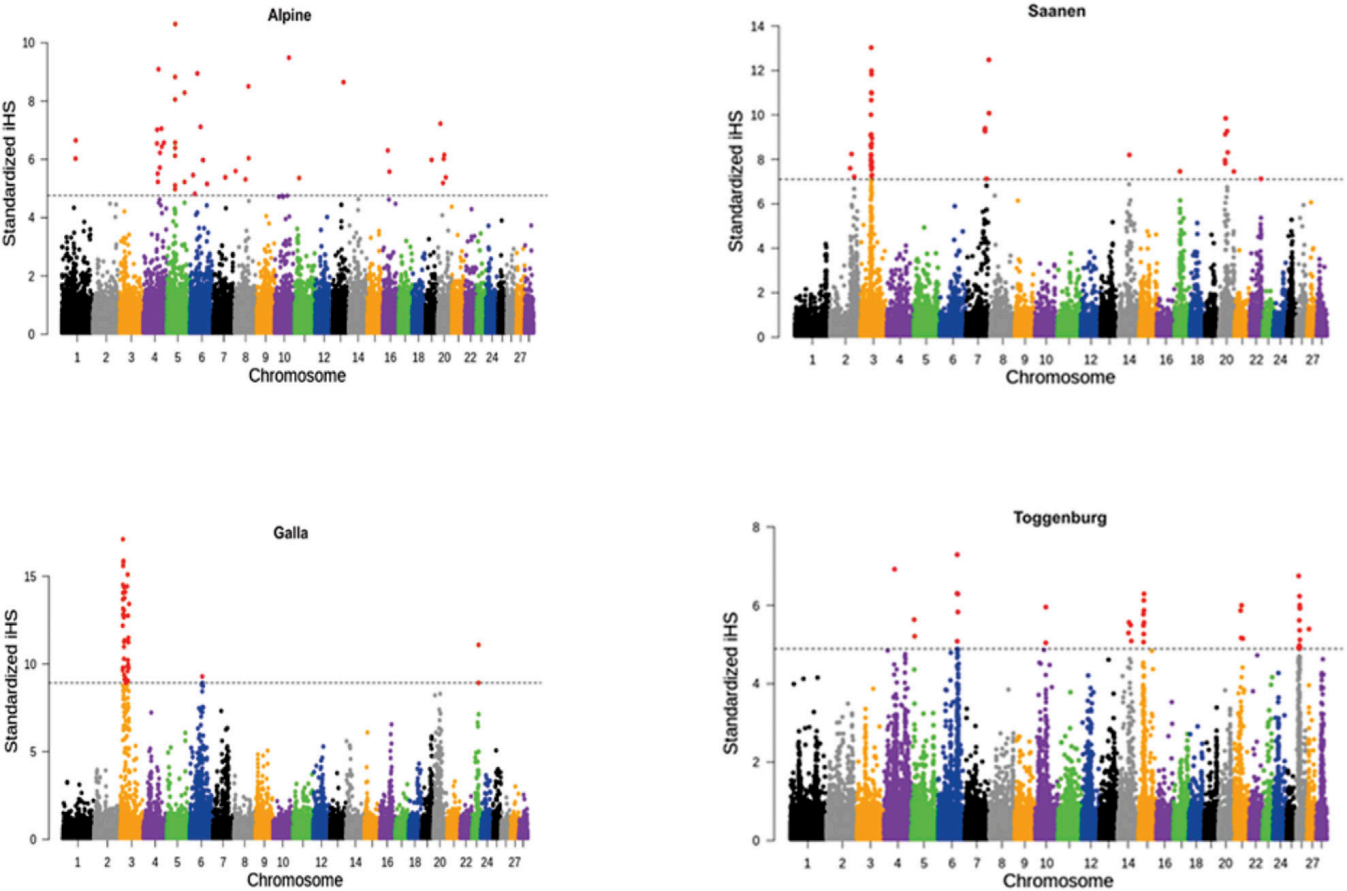
Genome-wide distribution of iHS values for Alpine, Galla, Saanen, and Toggenburg goat breeds. The dashed line represents top 0.01 percentile distribution of iHS values.

Figure 1 shows the genome-wide distribution of iHS values across the genome for each goat breed. Using the FDR <0.01 cutoff (as described earlier) for each breed, 27, 32, 24, and 27 signatures of selection in Alpine, Galla, Saanen, and Toggernburg were found, respectively. The regions in all the breeds were not uniformly distributed across the genome. Out of these regions (within breed), only selective sweeps on different chromosomes passed the clustering conditions for describing candidates for strong selection. A strong selective sweep was observed on chromosome 3 for both Galla and Saanen breeds, while in the Alpine breed, chromosomes 4 and 5 had a stronger signal than the other chromosomes. The strongest candidate selective sweep region in Toggenburg was on chromosomes 6, 15, and 26.

**FIGURE 2:**
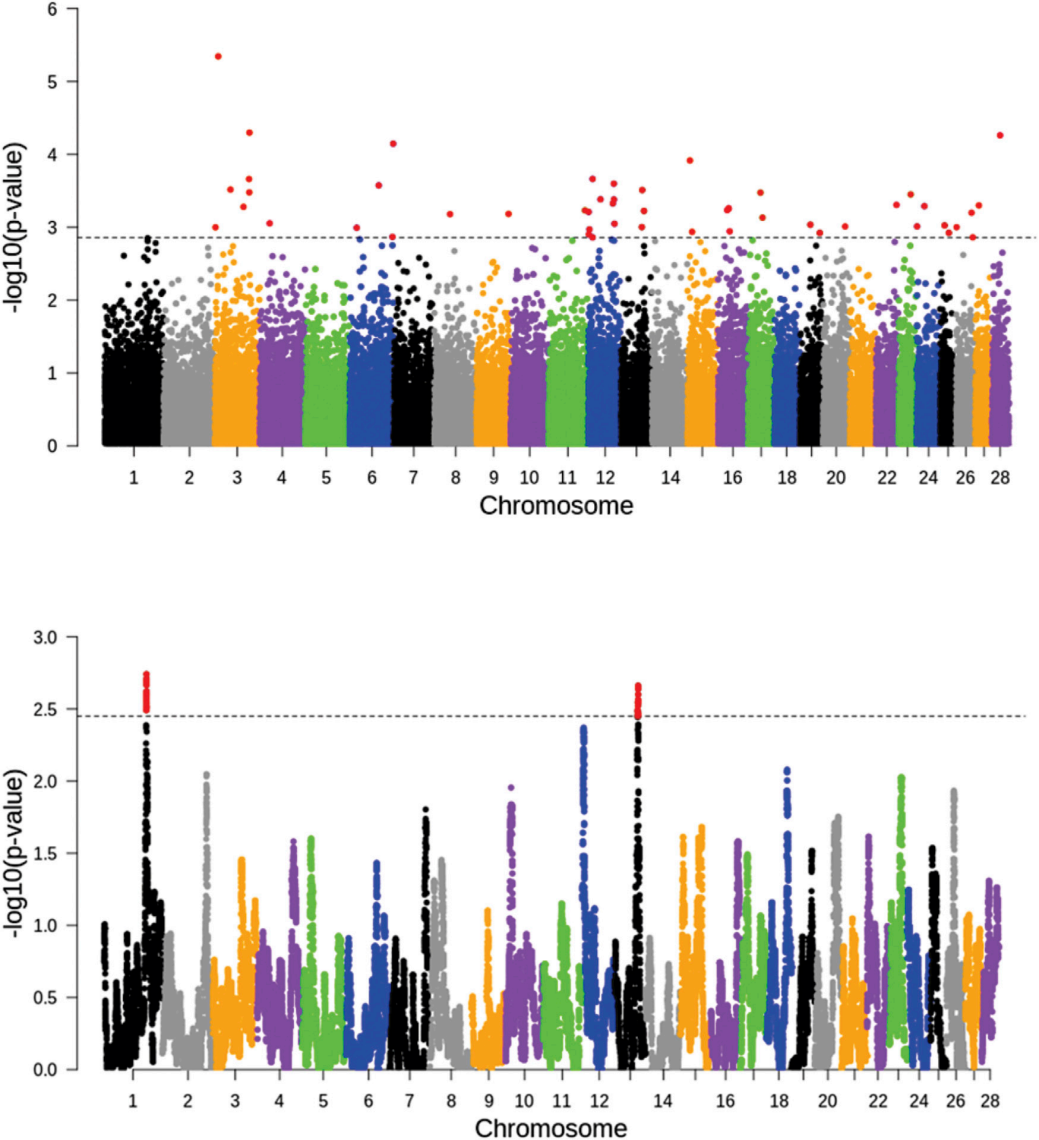
Distribution of FLK (top) and hapFLK (bottom) values with a dashed line representing a cutoff threshold of 0.01 percentile.

The FLK and hapFLK analyses detected a total of 100 signatures of selection in the four breeds (Figure 2). The FLK analyses detected regions of putative selection signature regions, notably with strong signals at chromosomes 3, 6, 12, 13, 16, and 26. While hapFLK revealed two putative genomic regions under selection in chromosome 1 and chromosome 13. Only the genomic region in chromosome 13 was detected by both FLK and hapFLK.

**FIGURE 3:**
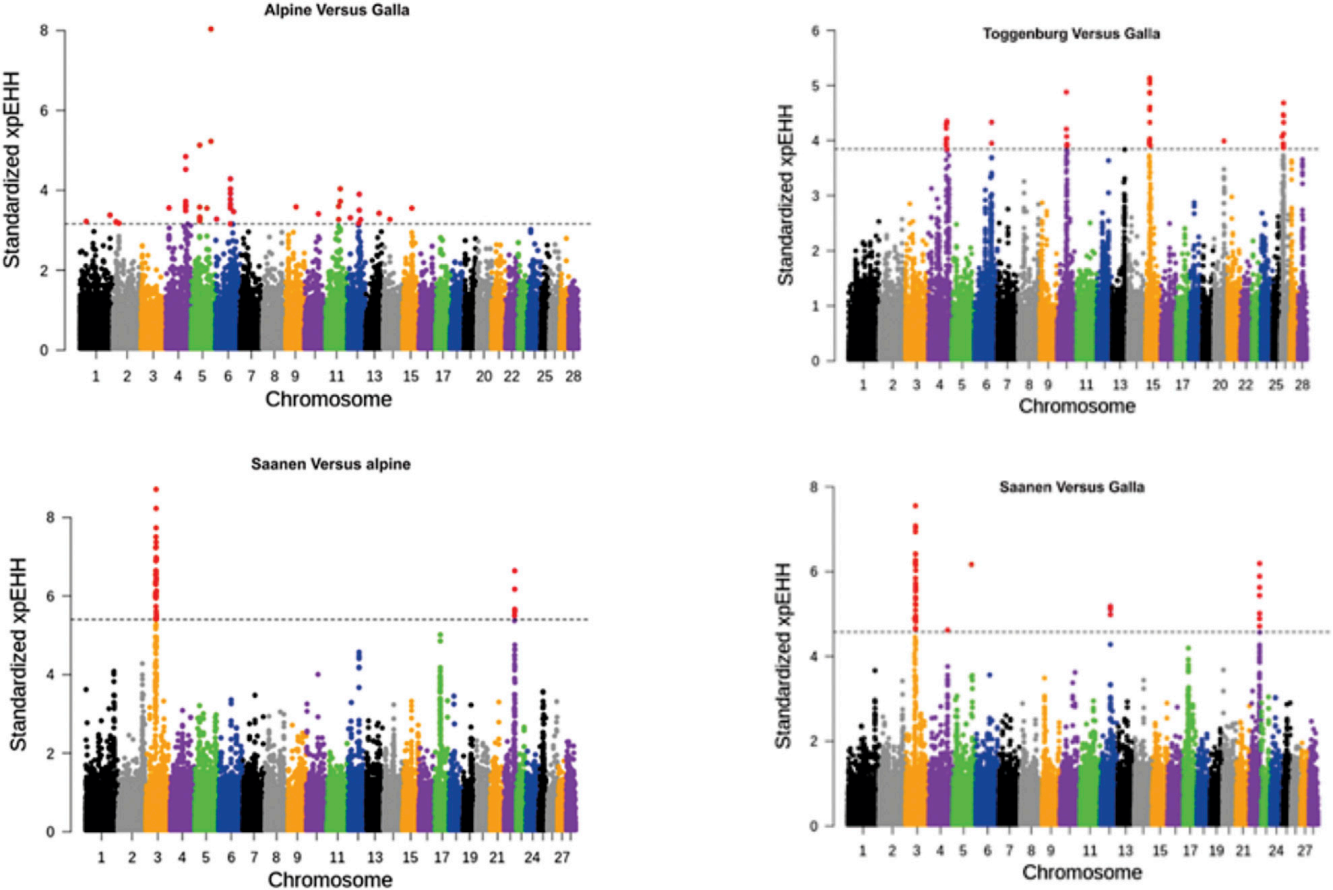
Distribution of standardized XP-EHH scores comparing amongst goat breeds. The dashed lines represent a cutoff threshold of 0.01 percentile of XP-EHH scores.

To identify loci under selection between two breeds, the XP-EHH was calculated for the two breeds separately, integrated over recombination distance, and performing comparison at last. Using the cutoff threshold of 0.01 percentile (as described earlier), several significant selection sweeps were detected. The Manhattan plot of the log-transformed XP-EHH values for different comparisons are presented in Figure 3. Chromosomes 3 and 22 were detected as the strongest signals for selection between Saanen and Galla and in Saanen vs. Alpine, consistent with chromosomes 3 in the iHS test for the Saanen breed. A significant region on chromosome 4 was detected in Alpine vs. Galla and Toggenburg vs. Galla.

### Putative Regions, Genes Identified, and Enrichment of Putative Selective Signatures

Although all the aforementioned tests detected candidate regions, genomic regions that passed the cutoff threshold (top genomic regions) (Supplementary Table S1), located within the same chromosomal position, and were within the intersection of the multiple-selective signal were considered the overlapping selection signature regions **(** Supplementary Table S2 **)**. The intersect analysis resulted in putative selection signature regions on chromosomes 3 (41.0–53.0 Mb), 4 (94.0–95.0 Mb), 10 (47.0–48.0 Mb), 13 (57.0–62.0 Mb), 15 (22.0–26.0 Mb), 22 (50.0–51.0 Mb), 26 (16.0–19.0 Mb), and 27 (9.0–10.0 Mb). Most of the putative regions were found to overlap significantly with the iHS and XP-EHH analyses on chromosomes 3, 4, 10, 15, 22, and 26. Comparing the genomic locations, there is limited or no overlap between the different breeds. For example, intersect between XP-EHH and iHS detected 47 genes on chromosome 22 (50.0–51.0 Mb) for the Saanen breed.

Within the overlapped selection signature regions identified, a list of genes was used to perform separate functional analyses using DAVID with default settings on the human gene set (*Homo sapiens*). A few of the candidate genes were identified, which may be playing a role in regulating immune responses include *HYAL1*, *HYAL2*, *HYAL3*, *PCK1*, *SFRP1*, and *DAG1*, among others. Supplementary Table S2 shows intersected selection regions detected and genes associated with the identified regions. Functional analysis of the genes from the intersected regions generated significant (*p* < 0.05) Gene Ontology (GO) of 36 biological process (BP) terms (Supplementary Table S3), 10 cellular component (CC) terms (Supplementary Table S4), and eight molecular function (MF) terms (Supplementary Table S5 **)**, and seven significant (*p* < 0.05) KEGG pathways were enriched (Supplementary Table S6).

The biological processes enriched were associated to cellular response to interleukin-1, cellular response to UV-B and hyaluronan catabolic process, cellular response to tumor necrosis factor, and response to antibiotics*,* among other GO terms. The cellular components (CC) include dendritic spine, extracellular exosome, midbody, basement membrane, and glutamategic synapse, among others. Whereas, molecular functions (MFs) were related to hyalurononglucosaminidase activity, GDP binding, GTP binding, hyaluronoglucuronidase activity, lipid binding, guanyl nucleotide binding, methyltransferase activity, and G-protein beta/gamma-subunit complex binding. The glycosaminoglycan degradation, arrhythmogenic right ventricular cardiomyopathy, axon guidance, autophagy–yeast, cAMP signaling pathway, parathyroid hormone synthesis, secretion and action, and pathways in cancer were among the enriched KEGG pathways.

## Discussion

### Selection Signatures

Detecting recent positive selection signatures in domesticated animals that have gone through both artificial and natural selection can provide information on genomic sites that can contribute to the identification of beneficial mutations and underlying biological pathways for economically important traits. Several methods have been used for the detection of selection signatures in various domestic animal species. The two commonly used approaches are by considering the regions of low genetic diversity within a population (linkage disequilibrium-based methods) and by investigating genomic areas that display high levels of differentiation among populations (population differentiation-based methods). Both approaches are able to detect signals that are a result of rapid fixation of initially rare variants (hard sweeps). The statistical power to identify selection signatures may differ among the methods. However, linkage disequilibrium-based methods are more powerful than population differentiated-based methods (Jacobs et al., 2016). These methods have been used in cattle (Cheruiyot et al., 2018; Tijjani et al., 2019), goats (Henkel et al., 2019; Islam et al., 2019), and sheep (Saravanan et al., 2021; Zhang et al., 2021). To enhance the accuracy of selection sweep detection and also to exclude unknown biasness, it is essential to combine various statistical approaches (Qanbari and Simianer, 2014).

The aim in this study was to detect selection signatures in the genome of selected four goat breeds in Kenya (Alpine, Galla, Saanen, and Toggenburg) using high-density genotypes (47,663 SNPs). Both linkage disequilibrium-based methods (iHS, XP-EHH, and hapFLK) and population differentiation-based method (FLK) were used in the analyses. The combination of XP-EHH, FLK, and hapFLK in a two-population sample comparison permits a slight improvement of power, showing that these various statistics capture patterns in the data differently (Fariello et al., 2013).

The application of FLK and hapFLK approaches in this study was because these methods have been used successfully in goat (Bertolini et al., 2018) and in sheep data (Fariello et al., 2014). These methods increase the power of signature of selection detection and also allow the detection of soft or incomplete selective sweeps. However, in this study, FLK and hapFLK detected very few intersect genomic regions with other analyses, and this is probably due to the FDR threshold applied and the small population sample size.

Although iHS and XP-EHH are both LD-based methods, according to Sabeti et al. (2007), the XP-EHH test uses a population comparison approach, while iHS requires haplotypes per individual of one population to detect selection (Voight et al., 2006). Cross-population-extended haplotype homozygosity is based on iHS and EHH, and calculation between and not within breeds makes the main difference (Sabeti et al., 2007).

The intersect analysis detected various putative selective regions. However, differentiating between true signatures of selection and those arising from admixture and genetic drift remain the most challenging task (Akey et al., 2002). Most of the putative regions were found to overlap significantly with the iHS and XP-EHH analyses on chromosomes 3, 4, 10, 15, 22, and 26. The presence of the overlap between the two analyses may be explained by the increased power of iHS in not having a good reference population in this study (Voight et al., 2006) and of XP-EHH in which alleles have increased in frequency to the point of fixation or near fixation in one of the populations (Sabeti et al., 2007). However, only one putative region on chromosome 13 (61.0–62.0 Mb) intersected between hapFLK and FLK. The lack of overlap for the top putative regions probably can be due to hapFLK being designed to mostly detect regions with migration and bottlenecks (Fariello et al., 2013). Furthermore, the haplotypes in these regions are probably not to be selected for in the corresponding breeds since hapFLK considers population stratification. The results also suggest that selection on those regions most closely did not resemble classic sweeps. Essentially, the results in this study were drawn from the analyses of Illumina GoatSNP50 BeadChip genotyping data. It is very possible that some key genome regions might not have been detected because the genome coverage and ascertainment bias of the tool was toward pure dairy goats, Saanen and Alpine, among others, which were included in the panel discovery (Tosser-Klopp et al., 2014). Worth noting, according to Waineina et al. (2021), the breeds in this study were admixed at different levels. Therefore, further research is recommended using whole-genome deep sequencing to build up the findings from this study. A further study will represent the genetic diversity of a series of local populations, resulting in the availability of large amount of genetic information.

Although the history of the selected goats in this study has witnessed founding events and introgression, demographic events most likely have influenced the pattern of the selected goats’ genome diversity (Waineina et al., 2021). It is challenging to differentiate between the effects of natural selection and demographic events. In addition, the identification of true signatures is complicated and of great concern because of false-positive arising from selection signatures (Gouveia et al., 2014). Estimating the degree of such biasness on selection signature analyses, fewer efforts have been applied to date. In this study, a stringent percentile (0.01) was applied for all the approaches to limit possible false-positives.

Comparing the genomic locations, there is limited or no overlap between different breeds. This showed that most of the selected regions were breed-specific and contained several genes reflecting definite phenotypic evolutions under various selection objectives or adaptations to the local environments. The iHS and XP-EHH analyses intersect detected candidate regions mostly in Saanen and Galla breeds on chromosomes 3 (46.0–53.0 Mb). The reasons might be Saanen and Galla breed alleles have almost reached the point of fixation, which is in agreement with Waineina et al. (2021) findings that the two breeds were less admixed than the other breeds and are managed in seclusion from other breeds in this study. Additionally, random genetic drift and natural selection for adaptation to their environment might have contributed to the detection of strong signals in candidate selection signatures.

In general, most of the selected regions were elusive and breed-specific, as expected for complex traits under selection. In this study, the forces stirring selection in the genome of various goat breeds may be connected with adaptation to tropical environments, such as disease and parasite resistance and the capability to perform in the harsh environment with limited feed and water resources. The identified candidate genes within the putative selective signatures associated with specific biological, molecular, and cellular pathways and functions may be shaping the genomic architecture of selected goat breeds in this study for survival in the harsh environment.

### Candidate Genes Associated With Selection Signatures

Interestingly, the three breeds (Saanen, Toggenburg, and Alpine) were introduced in Kenya to improve milk production of the indigenous goats, whereas the Galla goat population has been maintained as a pure breed in the government breeding station under artificial selection for many generations. Galla goat is known for its potential for meat, milk production, and survives better even under harsh tropical conditions.

Genomic analyses on four goat breeds using various approaches detected a number of putative regions of selection connected to economic traits: milk production, growth, disease resistance, and adaptation to stress from heat, parasite, and feeds. These regions harbored a number of candidate genes with various cellular, molecular, and biological functions for better production, reproduction, and adaptation to a wide range of environments and with special characteristics. This is because rather than a single candidate gene, a composite network of genes is known to control the adaptation processes to environmental challenges. A candidate gene is a gene that is accountable for a considerable amount of genetic variation of a trait (Moioli et al., 2007).

According to Zonaed Siddiki et al. (2020), over 271 candidate genes have been detected in goats, which impact the metabolism, physiological pathway, and expression of phenotypic characteristics. These genes play vital roles in economically important traits such as growth, reproduction, milk, and disease resistance. The biological pathways and genes identified within the candidate selection signatures further permit to categorize the diversity of selection pressure having molded the genome of goat breeds in Kenya.

The study identified genes (*HYAL1*, *HYAL2*, *HYAL3*, *PCK1*, *SFRP1*, and *MST1R*) associated with the biological process that regulates the immune system of goats: cellular response to interleukin-1, cellular response to UV-B, response to antibiotics, viral entry into the host, and response to virus, etc. In most tropical countries, diseases are the main challenges of productivity in goats. Some of the common diseases that affect goats in the tropical countries are pneumonia, helminthiasis, pox, ectoparasite, fever, anorexia, and alopecia, *etc*. The identification of multiple genes related to disease resistance would seem to propose that the trait is under intense natural selection pressure in the tropically adapted goat breed in their local environment. According to Kim et al. (2016), adaptation of indigenous Barki goats and sheep to hot arid environments may probably be a result of a complex network of genes. This is in agreement with Lv et al. (2014) findings that adaptation to a certain environment is a result of the interaction of various complex traits that are controlled by many genes and also found that due to weak selection acting on various regions across the genome, selection for complex traits leave behind limited or no classic footprints of selection (signatures of selection) (Kemper et al., 2014).

The genes identified for hypoxic adaptation are *MST1*, *PCK*, and *SFRP1*. They are responsible for cellular response to hypoxia biological processes. Hypoxia is caused by the low oxygen availability of high-altitude regions. The three study areas have an altitude of 1830–2210, 1460–1710, and 1166 m above the sea level. This is one of the most important environmental challenges to envisage animals. It enforces severe constraints on aerobic metabolism and causes high-altitude illness. The identification of the *MSTI* gene in the Saanen breed elucidates the importance of breeding Saanen for adaptive capability for a high-altitude environment. Saanen was introduced into the study area for crossbreeding with the indigenous goats; therefore, with time, the breed has adapted to the tropical environment stressors. It is important for an in-depth study at high resolution to identify more and precise genes associated with tropical adaptation and production in goat breeds in Kenya.

Candidate genes that might be involved in goat milk production identified according to biological processes are the negative regulation of gluconeogenesis (*LEPR* and *MST1*) and regulation of calcium ion transport (*GNAI2* and *RHOA*)*.* The *LEPR* gene regulates function that takes place in the liver. The liver is the major site for the increase of lipid β-oxidation and increased glycogenesis in early lactation, thus affecting blood glucose regulation and milk yield (Bauman and Currie, 1980). This biological pathway is vital in goat breeds in Kenya to maintain adequate energy production and activity in their harsh environment, especially during the lactation period. The *LEPR* gene is also related to hypoxic in Tibetan goats (Jin et al., 2020) and reproductive and fatness traits in goats and sheep (Alim et al., 2019; Gunawan et al., 2019). The parathyroid hormone synthesis, secretion, and action of the KEGG pathway is associated with *GNAI2*, *PDE4B*, and *RHOA* genes identified in this study. According to Ma et al. (2021), phosphodiesterase 4B (*PDE4B*) is one of the genes that control milk production traits in dairy cattle. Parathyroid glands play an important role in regulation of calcium balance in the body. Calcium is vital in milking goats, and its major biological function is bone formation. Parturition in goat is connected to sustained increase in secretion of the parathyroid hormone-related protein into both milk and plasma (Ratcliffe et al., 1992). These results show that artificial selection has played a vital role to shape the genome of the goat breeds in the study.

## Conclusion

This is the first study to identify candidate regions for selection signatures in the genome of four goat breeds in Kenya. The study identified several putative genomic regions and genes regulating immune responses (e.g., *HYAL1* and *HYAL3*), milk production (e.g., *LEPR* and *PDE4B*), and adaptation (e.g., *MST1* and *PCK*) that play an important role in underlying adaption to various environmental conditions in the selected Kenyan goat breeds. The results show that the diversity of selection forces has likely molded the genome of the goat breeds. Moreover, the results provide knowledge of the conservation and utilization of these goat genetic resources. In-depth research is necessary to further confirm and refine the results reported in this study by including comprehensive whole-genome deep sequencing and more samples, so that precise genes connected with adaptation and production in goat breeds in Kenya can be detected.

## Data Availability

The datasets presented in this study can be found in online repositories. The names of the repository/repositories and accession number(s) can be found in the article/Supplementary Material. Data availability to be accessible from the Mendeley Digital Repository (https://doi.org/10.17632/hhb9rhdzzt.1/).

## References

[B1] Ahuya C. O. Ojango J. M. K. Mosi R. O. Peacock C. P. Okeyo A. M. (2009). Performance of Toggenburg Dairy Goats in Smallholder Production Systems of the Eastern Highlands of Kenya. Small Ruminant Res. 83, 7–13. 10.1016/j.smallrumres.2008.11.012

[B2] Akey J. M. Zhang G. Zhang K. Jin L. Shriver M. D. (2002). Interrogating a High-Density SNP Map for Signatures of Natural Selection. Genome Res. 12, 1805–1814. 10.1101/gr.631202 12466284 PMC187574

[B3] Alim M. A. Hossain M. M. K. Nusrat J. Rubaya M. Salimullah M. Shu-Hong Z. (2019). Genetic Effects of Leptin Receptor (LEPR) Polymorphism on Litter Size in a Black Bengal Goat Population. Anim. Biol. 69 (4), 411–420. 10.1163/15707563-00001079

[B4] Ashburner M. Ball C. A. Blake J. A. Botstein D. Butler H. Cherry J. M. (2000). Gene Ontology: Tool for the Unification of Biology. Nat. Genet. 25, 25–29. 10.1038/75556 10802651 PMC3037419

[B5] Bauman D. E. Currie W. B. (1980). Partitioning of Nutrients during Pregnancy and Lactation: A Review of Mechanisms Involving Homeostasis and Homeorhesis. J. Dairy Sci. 63 (9), 1514–1529. 10.3168/jds.S0022-0302(80)83111-0 7000867

[B6] Bertolini F. Servin B. Servin B. Talenti A. Rochat E. Kim E. S. (2018). Signatures of Selection and Environmental Adaptation across the Goat Genome Post-Domestication. Genet. Sel Evol. 50, 1–24. 10.1186/s12711-018-0421-y 30449276 PMC6240954

[B7] Bett R. C. Kosgey I. S. Kahi A. K. Peters K. J. (2011). Definition of Breeding Objectives and Optimum Crossbreeding Levels for Goats in the Smallholder Production Systems. Small Ruminant Res. 96, 16–24. 10.1016/j.smallrumres.2010.11.008

[B8] Bolormaa S. Hayes B. J. Hawken R. J. Zhang Y. Reverter A. Goddard M. E. (2011). Detection of Chromosome Segments of Zebu and Taurine Origin and Their Effect on Beef Production and Growth. J. Anim. Sci. 89, 2050–2060. 10.2527/jas.2010-3363 21297063

[B9] Bonhomme M. Chevalet C. Servin B. Boitard S. Abdallah J. Blott S. (2010). Detecting Selection in Population Trees: The Lewontin and Krakauer Test Extended. Genetics 186, 241–262. 10.1534/genetics.110.117275 20855576 PMC2940290

[B10] Brito L. F. Kijas J. W. Ventura R. V. Sargolzaei M. Porto-Neto L. R. Cánovas A. (2017). Genetic Diversity and Signatures of Selection in Various Goat Breeds Revealed by Genome-Wide SNP Markers. BMC Genomics 18, 229. 10.1186/s12864-017-3610-0 28288562 PMC5348779

[B11] Chang C. C. Chow C. C. Tellier L. C. Vattikuti S. Purcell S. M. Lee J. J. (2015). Second-Generation PLINK: Rising to the challenge of Larger and Richer Datasets. Gigascience 4, 7. 10.1186/s13742-015-0047-8 25722852 PMC4342193

[B12] Charlesworth B. (2007). A Hitch-Hiking Guide to the Genome: A Commentary on 'The Hitch-Hiking Effect of a Favourable Gene' by John Maynard Smith and John Haigh. Genet. Res. 89, 389–390. 10.1017/S0016672308009580 18976526

[B13] Chenyambuga S. W. Hanotte O. Hirbo J. Watts P. C. Kemp S. J. Kifaro G. C. (2004). Genetic Characterization of Indigenous Goats of Sub-Saharan Africa Using Microsatellite DNA Markers. Asian Australas. J. Anim. Sci. 17, 445–452. 10.5713/ajas.2004.445

[B14] Cheruiyot E. K. Bett R. C. Amimo J. O. Zhang Y. Mrode R. Mujibi F. D. N. (2018). Signatures of Selection in Admixed Dairy Cattle in Tanzania. Front. Genet. 9, 607. 10.3389/fgene.2018.00607 30619449 PMC6305962

[B17] FAO (2012). “Phenotypic Characterization of Animal Genetic Resources,” in FAO Animal Production and Health Guidelines (Rome: Food and Agriculture Organization of the United Nations), 142.

[B18] FAO STAT (2018). Livestock Population. Available at: http://www.fao.org/faostat/en/#data/QA (Accessed December 2021).

[B19] Fariello M.-I. Servin B. Tosser-Klopp G. Rupp R. Moreno C. Cristobal M. S. (2014). Selection Signatures in Worldwide Sheep Populations. PLoS One 9, e103813. 10.1371/journal.pone.0103813 25126940 PMC4134316

[B20] Fariello M. I. Boitard S. Naya H. SanCristobal M. Servin B. (2013). Detecting Signatures of Selection through Haplotype Differentiation Among Hierarchically Structured Populations. Genetics 193, 929–941. 10.1534/genetics.112.147231 23307896 PMC3584007

[B21] Gautier M. Vitalis R. (2012). Rehh: An R Package to Detect Footprints of Selection in Genome-Wide SNP Data from Haplotype Structure. Bioinformatics 28, 1176–1177. 10.1093/bioinformatics/bts115 22402612

[B63] Gautier M. Naves M. (2011). Footprints of Selection in the Ancestral Admixture of a New World Creole Cattle Breed. Mol. Ecol. 20 (15), 3128–3143. 10.1111/j.1365-294X.2011.05163.x 21689193

[B23] Gouveia J. J. D. S. Silva M. V. G. B. D. Paiva S. R. Oliveira S. M. P. D. (2014). Identification of Selection Signatures in Livestock Species. Genet. Mol. Biol. 37 (2), 330–342. 10.1590/s1415-47572014000300004 25071397 PMC4094609

[B24] Gunawan A. Pramukti F. W. Listyarini K. Abuzahra M. A. Jakaria J. Sumantri C. (2019). Novel Variant in the Leptin Receptor (LEPR) Gene and its Association with Fat Quality, Odour and Flavour in Sheep. J. Indonesian Trop. Anim. Agric. 44 (1), 1–9. 10.14710/jitaa.44.1.1-9

[B25] Henkel J. Saif R. Jagannathan V. Schmocker C. Zeindler F. Bangerter E. (2019). Selection Signatures in Goats Reveal Copy Number Variants Underlying Breed-Defining Coat Color Phenotypes. Plos Genet. 15, e1008536. 10.1371/journal.pgen.1008536 31841508 PMC6936872

[B26] Islam R. Li Y. Liu X. Berihulay H. Abied A. Gebreselassie G. (2019). Genome-Wide Runs of Homozygosity, Effective Population Size, and Detection of Positive Selection Signatures in Six Chinese Goat Breeds. Genes 10, 938. 10.3390/genes10110938 PMC6895971 31744198

[B27] Jacobs G. S. Sluckin T. J. Kivisild T. (2016). Refining the Use of Linkage Disequilibrium as a Robust Signature of Selective Sweeps. Genetics 203, 1807–1825. 10.1534/genetics.115.185900 27516617 PMC4981279

[B28] Jin M. Lu J. Fei X. Lu Z. Quan K. Liu Y. (2020). Selection Signatures Analysis Reveals Genes Associated with High-Altitude Adaptation in Tibetan Goats from Nagqu, Tibet. Animals 10 (9), 1599. 10.3390/ani10091599 PMC7552128 32911823

[B29] Kanehisa M. Goto S. Sato Y. Furumichi M. Tanabe M. (2012). KEGG for Integration and Interpretation of Large-Scale Molecular Data Sets. Nucleic Acids Res. 40, D109–D114. 10.1093/nar/gkr988 22080510 PMC3245020

[B30] Kemper K. E. Saxton S. J. Bolormaa S. Hayes B. J. Goddard M. E. (2014). Selection for Complex Traits Leaves Little or No Classic Signatures of Selection. BMC Genomics 15, 246. 10.1186/1471-2164-15-246 24678841 PMC3986643

[B31] Kim E.-S. Elbeltagy A. R. Aboul-Naga A. M. Rischkowsky B. Sayre B. Mwacharo J. M. (2016). Multiple Genomic Signatures of Selection in Goats and Sheep Indigenous to a Hot Arid Environment. Heredity 116, 255–264. 10.1038/hdy.2015.94 26555032 PMC4806575

[B32] Kim Y. Stephan W. (2002). Detecting a Local Signature of Genetic Hitchhiking along a Recombining Chromosome. Genetics 160, 765–777. 10.1093/genetics/160.2.765 11861577 PMC1461968

[B33] Kiura J. N. Abdi Yakub G. Migwi P. K. Ondiek J. O. (2020). Performance and Genotypes of Dairy Goats in Kenya: Lessons Learnt and the Need to Move beyond Donor Introductions. J. Agric. Sci. Technol. A 10, 128–137. 10.17265/2161-6256/2020.03.003

[B34] Luikart G. Gielly L. Excoffier L. Vigne J.-D. Bouvet J. Taberlet P. (2001). Multiple Maternal Origins and Weak Phylogeographic Structure in Domestic Goats. Proc. Natl. Acad. Sci. U.S.A. 98, 5927–5932. 10.1073/pnas.091591198 11344314 PMC33315

[B35] Lv F.-H. Agha S. Kantanen J. Colli L. Stucki S. Kijas J. W. (2014). Adaptations to Climate-Mediated Selective Pressures in Sheep. Mol. Biol. Evol. 31, 3324–3343. 10.1093/molbev/msu264 25249477 PMC4245822

[B36] Ma Y. Khan M. Z. Xiao J. Alugongo G. M. Chen X. Chen T. (2021). Genetic Markers Associated with Milk Production Traits in Dairy Cattle. Agriculture 11, 1018. 10.3390/agriculture11101018

[B37] McVean G. (2007). The Structure of Linkage Disequilibrium Around a Selective Sweep. Genetics 175, 1395–1406. 10.1534/genetics.106.062828 17194788 PMC1840056

[B38] Moioli B. D’Andrea M. Pilla F. (2007). Candidate Genes Affecting Sheep and Goat Milk Quality. Small Ruminant Res. 68, 179–192. 10.1016/j.smallrumres.2006.09.008

[B39] Naderi S. Rezaei H.-R. Taberlet P. Zundel S. Rafat S.-A. Naghash H.-R. (2007). Large-Scale Mitochondrial DNA Analysis of the Domestic Goat Reveals Six Haplogroups with High Diversity. PLoS One 2, e1012. 10.1371/journal.pone.0001012 17925860 PMC1995761

[B40] Ndeke A. Mutembei H. Tsuma V. Mutiga E. (2015). Reproductive Performance of the Galla and Toggenburg Goats and Their Crosses in Mwingi Sub-county of Kenya. J. Agric. Sci. Food Technol. 1, 78–83.

[B41] Onzima R. B. Upadhyay M. R. Doekes H. P. Brito L. F. Bosse M. Kanis E. (2018). Genome-Wide Characterization of Selection Signatures and Runs of Homozygosity in Ugandan Goat Breeds. Front. Genet. 9, 318. 10.3389/fgene.2018.00318 30154830 PMC6102322

[B42] Pillian B. Racokzi G. (1976). Breeding and Research. FAO/UNDP Sheep and Goat Development Project AG: DP/KENI71/527 Technical report 3, 1976.

[B43] Qanbari S. Simianer H. (2014). Mapping Signatures of Positive Selection in the Genome of Livestock. Livestock Sci. 166, 133–143. 10.1016/j.livsci.2014.05.003

[B44] Ratcliffe W. A. Thompson G. E. Caret A. D. Peaker M. (1992). Production of Parathyroid Hormone-Related Protein by the Mammary Gland of the Goat. J. Endocrinol. 133, 87–93. 10.1677/joe.0.1330087 1517711

[B45] Sabeti P. C. Varilly P. Varilly P. Fry B. Lohmueller J. Hostetter E. (2007). Genome-Wide Detection and Characterization of Positive Selection in Human Populations. Nature 449, 913–918. 10.1038/nature06250 17943131 PMC2687721

[B46] Saravanan K. A. Panigrahi M. Kumar H. Bhushan B. Dutt T. Mishra B. P. (2021). Genome-Wide Analysis of Genetic Diversity and Selection Signatures in Three Indian Sheep Breeds. Livestock Sci. 243, 104367. 10.1016/j.livsci.2020.104367

[B47] Scheet P. Stephens M. (2006). A Fast and Flexible Statistical Model for Large-Scale Population Genotype Data: Applications to Inferring Missing Genotypes and Haplotypic Phase. Am. J. Hum. Genet. 78, 629–644. 10.1086/502802 16532393 PMC1424677

[B48] Sherman B. T. Lempicki R. A. (2009). Systematic and Integrative Analysis of Large Gene Lists Using DAVID Bioinformatics Resources. Nat. Protoc. 4, 44–57. 10.1038/nprot.2008.211 19131956

[B49] Shivairo R. Matofari J. Muleke C. Migwi P. Lugairi E. (2013). Production Challenges and Socio-Economic Impact of Dairy Goat Farming Amongst Smallholder Farmers in Kenya. Food Sci. Qual. Manage. 17, 54–61.

[B50] Smith J. M. Haigh J. (1974). The Hitch-Hiking Effect of a Favourable Gene. Genet. Res. 23, 23–35. 10.1017/S0016672300014634 4407212

[B62] Taberlet P. Valentini A. Rezaei H. Naderi S. Pompanon F. Negrini R. (2008). Are Cattle, Sheep, and Goats Endangered Species?. Mol. Ecol. 17, 275–284. 10.1111/j.1365-294X.2007.03475.x 17927711

[B51] Tijjani A. Utsunomiya Y. T. Ezekwe A. G. Nashiru O. Hanotte O. (2019). Genome Sequence Analysis Reveals Selection Signatures in Endangered Trypanotolerant West African Muturu Cattle. Front. Genet. 10, 442. 10.3389/fgene.2019.00442 31231417 PMC6558954

[B52] Tosser-Klopp G. Bardou P. Bouchez O. Cabau C. Crooijmans R. Dong Y. (2014). Design and Characterization of a 52K SNP Chip for Goats. PloS one 9, e86227. 10.1371/journal.pone.0086227 24465974 PMC3899236

[B53] Voight B. F. Kudaravalli S. Wen X. Pritchard J. K. (2006). A Map of Recent Positive Selection in the Human Genome. Plos Biol. 4, e72. 10.1371/journal.pbio.0040072 16494531 PMC1382018

[B55] Waineina R. W. Ngeno K. Okeno T. O. Ilatsia E. D. (2021). Genetic Diversity and Population Structure Among Indigenous and Imported Goat Breeds in Kenya. GenResJ 2, 25–35. 10.46265/genresj.EQFQ1540

[B56] Wang X. Liu J. Zhou G. Guo J. Yan H. Niu Y. (2016). Whole-Genome Sequencing of Eight Goat Populations for the Detection of Selection Signatures Underlying Production and Adaptive Traits. Sci. Rep. 6, 38932. 10.1038/srep38932 27941843 PMC5150979

[B57] Weigand H. Leese F. (2018). Detecting Signatures of Positive Selection in Non-Model Species Using Genomic Data. Zoolog. J. Linn. Soc. 184, 528–583. 10.1093/zoolinnean/zly007

[B59] Zeder M. A. Hesse B. (2000). The Initial Domestication of Goats (*Capra H*) in the Zagros Mountains 10,000 Years Ago. Science 287, 2254–2257. 10.1126/science.287.5461.2254 10731145

[B60] Zhang Y. Xue X. Liu Y. Abied A. Ding Y. Zhao S. (2021). Genome-Wide Comparative Analyses Reveal Selection Signatures Underlying Adaptation and Production in Tibetan and Poll Dorset Sheep. Sci. Rep. 11, 1–12. 10.1038/s41598-021-81932-y 33510350 PMC7844035

[B61] Zonaed Siddiki A. M. A. M. Miah G. Islam M. S. Kumkum M. Rumi M. H. Baten A. (2020). Goat Genomic Resources: The Search for Genes Associated with its Economic Traits. Int. J. Genomics 2020, 1–13. 10.1155/2020/5940205 PMC7456479 32904540

